# The prevalence of pain and receipt of pain management in inpatient psychiatric settings in Ontario

**DOI:** 10.1177/20551029261458472

**Published:** 2026-07-05

**Authors:** Megan Bryson, Lynn Martin, Michel Bedard

**Affiliations:** 1 7890 Department of Health Sciences, Lakehead University, Thunder Bay, Canada

**Keywords:** pain, mental illness, prevalence, adults, health care

## Abstract

Pain is often underreported and undertreated among individuals with mental illness receiving health care services. Few studies have reported on prevalence of pain and pain management within psychiatric settings; this study aims to fill that gap. Secondary analysis of cross-sectional anonymized population-level data from the Ontario Mental Health Reporting System (N = 315,934 unique individuals) was conducted to identify associations between psychiatric diagnoses and recognition of pain management needs while controlling for factors previously linked to pain. Approximately 22% reported pain, yet only 49% had recognized pain management needs. Age under 65, financial trade-offs, heavy drinking, opiate use, self-reported trauma, PTSD, substance use disorder, and cognitive impairment were associated with increased odds of recognition of pain management needs, while admission from an institution or homelessness, repeated psychiatric admissions, substance use, health instability, and several psychiatric diagnoses were associated with decreased odds. These findings highlight the need for systematic recognition of identified pain within psychiatric care.

## Plain Language Summary

People with mental illness are more likely to experience pain, but do not always receive the care they need to manage it. Data on over 315,000 people who stayed in psychiatric hospitals in Ontario were analyzed to find out how common pain was and whether people were offered or received treatment for pain. About one in five people had pain, but fewer than half were offered or received pain management. Certain groups, for example, those who were under 65 years, had trauma or substance-related diagnoses were more likely to be offered or receive treatment, while treatment was less likely for those who were homeless, had multiple previous psychiatric admissions, had an intellectual or developmental disability, or had psychotic, bipolar, depressive, personality, or medication-induced disorders. More work is needed to ensure pain is properly recognized and treated in mental health settings.

## Introduction

Pain is a complex experience that affects quality of life and functional ability (Raja et al., 2020). Painful conditions and illness are the leading global cause of years lived with disability (Stubbs et al., 2014). In Canada, the Canadian Pain Task Force estimated 7.6 million individuals live with chronic pain (Health Canada, 2021). According to Health Canada (2021) this number is expected to increase by 17.5% from 2019 to 2030 due to population aging. By 2030 as many as 9.0 million Canadians could be living with chronic pain. While pain can be experienced by all demographic groups, certain populations are more vulnerable to pain and its detrimental consequences.

Individuals with mental illness are disproportionately affected by both acute and chronic pain (Johnston and Huckins, 2022; Public Health Agency of Canada, 2012; Onwumere et al., 2022), yet they often receive inadequate or inconsistent pain assessment and treatment (Burton et al., 2016; Viprey et al., 2021). Under-managed pain is often associated with several adverse outcomes and reduced quality of life (Proctor and Hirdes 2001; Raja et al., 2020; Robitaille et al., 2024). The dynamic interplay among pain and mental illness make it challenging to treat either condition independent of each other (Hirdes et al., 2010). Effective pain management is essential to overall health and well-being, but becomes particularly demanding when accompanied by comorbid mental illness.

Those diagnosed with mental illness often encounter significant barriers to accessing care (Defar et al., 2023) and receive fragmented care from multiple settings (Hirdes et al., 2020). Chronic pain has a negative effect on the persistence of psychiatric disorders, thus creating a cyclical relationship that complicates treatment outcomes (Stubbs et al., 2014). As such, addressing pain in the context of mental illness requires an integrated, multidisciplinary approach that considers both physical and psychological aspects (Stubbs et al., 2014; Sadath et al., 2023). Selecting the most appropriate technique or combination of techniques can be challenging for individuals who present with complex, chronic pain profiles. Consequently, effective pain management relies not only on clinical expertise but also on ongoing assessment and collaboration with individuals to tailor interventions that address both physical and psychosocial dimensions of pain (Chen et al., 2014; Knopp-Sihota et al., 2022; Onwumere et al., 2022). Underreporting of pain is linked to fragmented care systems and mental illness-related stigma, further complicating effective management (Hirdes et al., 2020; Stubbs et al., 2014). Stigma may also contribute to lack of attention paid to pain among persons with mental illness by clinicians – i.e., discrimination. Bias may also lead to reports of pain by those with mental illness or substance use histories being devalued or dismissed by clinicians (e.g., implicit bias theory, see Greenwald and Banaji, 1995).

While pain is more prevalent among persons with mental illness overall, research has shown that some diagnoses are associated with higher rates of pain than others (Hirdes et al., 2010; Johnston and Huckins, 2022). For example, some have suggested that 65% of individuals with depression report pain (Onwumere et al., 2022) and between 10% and 50% of people with post-traumatic stress disorder (PTSD) report chronic pain (Gasperi et al., 2021). Hirdes and colleagues (2010) reported that one third of persons with anxiety disorder in inpatient mental health settings reported daily pain. A meta-analysis revealed that approximately 28.9% of persons with bipolar disorder and one third of those with schizophrenia experienced pain (Stubbs et al., 2014). Estimates also suggest that 50-60% of individuals with substance use disorders experience chronic pain (Wyse et al., 2021) and are at a greater risk for under-managed pain than those without substance use disorders (Chang and Compton 2013).

Appropriate pain management for individuals with mental illness presents an array of challenges. Pain is a deeply subjective experience, and its recognition and management are often clouded by systemic, clinical, and social barriers (Sadath et al., 2023). Further, pain management among those with mental illness hold a complex pathway due to the interplay between psychological and physical health symptoms. For instance, certain mental illnesses can alter pain perceptions, expression and reporting, making it difficult to accurately assess and manage (Onwumere et al., 2022). Severe and persistent mental illnesses such as schizophrenia or bipolar disorder are associated with elevated pain thresholds or dampened affective responses which may lead to underreporting or misinterpretation of symptoms (Carrasco-Picazo et al., 2023; Stubbs et al., 2014). Additionally, stigma, fragmented care systems and provider hesitancy, especially around opioid prescription can further complicate pain management for those with mental illnesses (De Kleijn et al., 2022; Papadomanolakis-Pakis et al., 2021). Overall, these challenges represent key barriers to recognizing and effectively managing pain among this vulnerable population.

Despite increasing recognition of mental illness and pain comorbidities, there remain notable gaps in the literature. For example, many studies rely on small, non-representative samples which often focus on specific psychiatric diagnoses, which limits understanding of pain across populations with different types of mental illness (Johnston and Huckins, 2022). Further, research often reports on findings from outpatient or community-based health care settings, with limited attention to specialized mental health settings.

### Objectives

This study aimed to better understand pain prevalence and factors associated with recognition of pain management needs among individuals admitted to inpatient psychiatric settings. In particular, the intent was to examine whether patterns of association observed among persons with mental illness receiving general health care services are present in specialized psychiatric care settings.

## Methods

### Design and data source

This was a cross-sectional, observational study based on population-level secondary data from the Ontario Mental Health Reporting System (OMHRS), which is based on information from interRAI’s Mental Health (MH) assessment (Hirdes et al., 2000). The MH is a valid and reliable comprehensive assessment that collects information on individual demographics, diagnoses, health status, service use, and functioning (Hirdes et al., 2020). The study included the admission assessments of all adults aged 18 or older admitted to an inpatient psychiatric bed, unit, or facility in Ontario between October 1, 2005, and March 31, 2023 (*n* = 312,440).

As this study involved secondary analysis of anonymized data, it was granted an ethics waiver by the Lakehead University Research Ethics Board under Article 2.4 of the Tri-Council Policy Statement (Canadian Institutes of Health Research, Natural Sciences and Engineering Research Council of Canada, and Social Sciences and Humanities Research Council of Canada, 2022).

### Measures

#### Pain

The Pain Scale is embedded in the assessment and based on two items: presence and intensity of pain (Fries et al., 2001; Hirdes et al., 2020). The scale scores range from 0 = No pain, 1 = Less than daily pain, 2 = Daily pain that is not severe, 3 = Daily severe pain, and 4 = Daily excruciating pain. A score of one or greater was used to indicate the presence of pain.

#### Recognition of pain management needs

The assessment provides a list of interventions and treatments offered to or received by the person, including pain management. Recognition of pain management needs was coded as “yes” if the assessment indicated that the person had been offered or received pain management, or if pain management had not yet been received but was scheduled to start.

#### Psychiatric diagnoses

The assessment includes a list of DSM-IV provisional psychiatric categories, each rated as 0 = Not present, 1 = Most important, 2 = Second most important, 3 = Third most important, 4 = Less important, or 5 = No provisional diagnoses. In this study, diagnoses were recoded into 1 = Present (rating = 1, 2, 3, or 4) and 0 = Not present (rating = 0 or 1).

#### Covariates

All covariates were drawn from items in the MH assessment and informed by review of literature pertaining to pain; these included personal (e.g., sex, age, place of residence before admission), social (e.g., partner status, financial trade-offs), and clinical (e.g., substance use, psychiatric history, medical multimorbidity) characteristics. Embedded scales related to cognition (i.e., Cognitive Performance Scale (CPS); Morris et al., 1994) and health instability (i.e., Changes in Health, End-stage Signs and Symptoms (CHESS) Scale; Hirdes et al., 2003) were also used; established cut-offs were used to indicate presence of cognitive impairment and health instability. The assessment also captures presence of five specific medical diagnoses (i.e., asthma, diabetes mellitus, hypothyroidism, migraine, and traumatic brain injury), with the option to manually enter additional diagnoses. As the team did not have access to the open-text diagnoses, responses to the five listed conditions were summed to create a multimorbidity variable; multimorbidity was coded as “yes” if the person had two or more medical diagnoses.

### Statistical analyses

All variables were described in terms of percentages for the full population, as well as by presence of pain. Pearson’s chi-square tests were used to compare the characteristics of those with and without pain. Bivariate logistic regression was used to explore the unadjusted relationships between covariates and receipt of pain management. Multicollinearity was assessed using correlation matrices and variance inflation factors (VIF); variables with VIF >5 were removed). A multivariate logistic regression model was constructed that took multicollinearity into account; model fit was assessed using −2 Log Likelihood, Akaike Information Criterion (AIC), Schwarz Criterion (SC), and Concordance statistic. All analyses were conducted in SAS 9.4. Statistical significance was set at *p* < 0.05.

## Results

### Population characteristics

Table 1 presents personal, social, and clinical characteristics for all persons admitted to inpatient psychiatry and by presence of pain. Overall, approximately 67.8% of individuals were aged 25–64 years, just over half were male (51.4%), and over two thirds reported not having a partner (70.5%). Most individuals had been admitted from either a private home (49.4%) or a hospital setting (27.8%), and relatively few had made financial trade-offs (e.g., between purchasing food and paying rent) in the previous month (5.1%). Approximately 58.3% had no psychiatric admissions in the two previous years. Most reported no alcohol consumption in the previous 2 weeks (72.1%) or substance use in the previous week (70.8%). The most prevalent psychiatric diagnoses were depressive disorders (16.9%), psychotic disorders (16.1%), and substance use disorders (13.7%); approximately 11.6% of individuals reported trauma. Multimorbidity and health instability were relatively low (3.3% and 12.8%, respectively), and most (68.7%) showed no signs of cognitive impairment.

**Table 1.** Population characteristics by pain status.

Characteristics	All *N* = 312,440 *N* (%)	No Pain *N* = 242,911 *N* (%)	Pain *N* = 69,529 *N* (%)	Test statistic and p-value
Age				χ2(df = 3) = 7134.40; *p* < 0.0001
Under 25 years	51,363 (16.5%)	45,502 (18.7%)	5,861 (8.4%)	
25-44 years	113,137 (36.2%)	91,144 (37.5%)	21,993 (19.4%)	
45-64 years	98,514 (31.5%)	71,633 (29.5%)	26,881 (38.7%)	
65+ years	49,294 (15.8%)	34,535 (14.2%)	14,759 (21.2%)	
Sex				χ2(df = 2) = 1084.69; *p* < 0.0001
Male	160,474 (51.4%)	128,592 (52.9%)	31,882 (45.9%)	
Female	151,834 (48.6%)	114,222 (47.0%)	37,612 (54.1%)	
Other	132 (0.04%)	97 (0.04%)	35 (0.1%)	
Has a partner (yes)	92,264 (29.5%)	68,873 (28.4%)	23,391(33.6%)	χ2(df = 1) = 726.94; *p* < 0.0001
Admitted from				χ2(df = 5) = 615.58; *p* < 0.0001
Private home	154,247 (49.4%)	121,100 (49.9%)	33,147 (47.6%)	
Congregated living	8,466 (2.7)	6,115 (2.5%)	2,351 (3.4%)	
Hospital setting	86,826 (27.8%)	68,523 (28.2%)	18,303 (26.3%)	
Homeless	7,646 (2.5%)	5,834 (2.4%)	1,812 (2.6%)	
Correctional facility	3,031 (1.0%)	2,438 (1.0%)	593 (0.8%)	
Other	6,697 (2.1%)	5,037 (2.1%)	1,660 (2.4%)	
Financial trade-offs in last month (yes)	15,773 (5.1%)	10,926 (4.4%)	4,847 (6.9%)	χ2(df = 1) = 686.34; *p* < 0.0001
Psychiatric admissions-last 2yrs				χ2(df = 2) = 131.64; *p* < 0.0001
None	182,021 (58.3%)	140,219 (57.7%)	41,802 (60.1%)	
1–2	97,269 (31.2%)	76,462 (31.5%)	20,807 (29.9%)	
3+	33,150 (10.6%)	26,230 (10.7%)	6,920 (9.9%)	
Highest # alcoholic drinks in single sitting				χ2(df = 3) = 349.19; *p* < 0.0001
None	225,167 (72.1%)	176,629 (72.7%)	48,538 (69.8%)	
1	12,442 (4.0%)	9,526 (3.9%)	2,916 (4.2%)	
2–4	29,041 (9.3%)	22,635 (9.3%)	6,406 (9.2%)	
5+	45,790 (14.7%)	34,121 (14.0%)	11,669 (16.8%)	
Substance use in the last week				
Inhalants	1,541 (0.5%)	1,194 (0.5%)	347 (0.5%)	χ2(df = 1) = 0.047; *p* = 0.8276
Hallucinogen	41,44 (1.3%)	3,329 (1.4%)	815 (1.2%)	χ2(df = 1) = 16.14; *p* < 0.0001
Cocaine	22,945 (7.3%)	16,836 (6.9%)	6,109 (8.8%)	χ2(df = 1) = 273.25; *p* < 0.0001
Stimulants	14,048 (4.5%)	10,429 (4.3%)	3,619 (5.2%)	χ2(df = 1) = 104.57; *p* < 0.0001
Opiates	16,802 (5.4%)	8,653 (3.5%)	8,149 (11.7%)	χ2(df = 1) = 7062.55; *p* < 0.0001
Cannabis	73,123 (23.4)	57,123 (23.5%)	16,000 (23.0%)	χ2(df = 1) = 7.70; *p* = .0056
Any substance	91,088 (29.2%)	68,401 (28.1%)	22,687 (32.6%)	χ2(df = 1) = 521.89; *p* < 0.0001
Reports trauma (yes)	36,074 (11.6%)	24,470 (10.0%)	11,604 (16.7%)	χ2(df = 1) = 2316.09; <0.0001
Psychiatric diagnoses				
Neurodevelopmental disorder	5,276 (1.7%)	4,425 (1.8%)	851 (1.2%)	χ2(df = 1) = 116.57; p < 0.0001
Psychotic disorder	50,304 (16.1%)	42,870 (17.6%)	7,434 (10.7%)	χ2(df = 1) = 1935.18; *p* < 0.0001
Bipolar disorder	23,967 (7.7%)	19,468 (8.0%)	4,499 (6.5%)	χ2(df = 1) = 181.33; *p* < 0.0001
Depressive disorder	52,685 (16.9%)	40,757 (16.8%)	119,28 (17.2%)	χ2(df = 1) = 5.48; *p* = 0.0193
Anxiety disorder	18,652 (6.0%)	14,347 (5.9%)	4,305 (6.2%)	χ2(df = 1) = 7.75; *p* = 0.0051
Obsessive compulsive disorder	2,546 (0.8%)	2,126 (0.9%)	420 (0.6%)	χ2(df = 1) = 49.01; *p* < 0.0001
Post traumatic stress disorder	13,082 (4.2%)	9,318 (3.8%)	3,764 (5.4%)	χ2(df = 1) = 335.81; *p* < 0.0001
Dissociative disorder	222 (0.07%)	164 (0.07%)	58 (0.1%)	χ2(df = 1) = 1.92; *p* = 0.1652
Somatic disorder	682 (0.2%)	369 (0.2%)	313 (0.5%)	χ2(df = 1) = 220.83; *p* < 0.0001
Eating disorder	2,042 (0.7%)	1,583 (0.6%)	459 (0.7%)	χ2(df = 1) = 0.01; *p* = 0.8068
Elimination disorder	48 (0.02%)	28 (0.01%)	20 (0.02%)	χ2(df = 1) = 10.45; *p* = 0.0012
Sleep disorder	376 (0.1%)	263 (0.1%)	113 (0.2%)	χ2(df = 1) = 13.24; *p* = 0.0003
Sexual disorder	50 (0.02%)	39 (0.01%)	11 (0.01%)	χ2(df = 1) = 0.00; *p* = 0.9656
Gender disorder	245 (0.1%)	216 (0.1%)	29 (0.04%)	χ2(df = 1) = 15.97; *p* < 0.0001
Impulse disorder	1,398 (0.5%)	1,146 (0.5%)	252 (0.3%)	χ2(df = 1) = 14.79; *p* = 0.0001
Substance use disorder	42,809 (13.7%)	32,291 (13.2%)	10,518 (15.1%)	χ2(df = 1) = 152.92; *p* < 0.0001
Neurocognitive disorder	9,637 (3.1%)	6,713 (2.7%)	2,924 (4.2%)	χ2(df = 1) = 376.37; *p* < 0.0001
Personality disorder	15,412 (4.9%)	11,972 (4.9%)	3,440 (1.4%)	χ2(df = 1) = 0.052; *p* = 0.8328
Paraphilic disorder	109 (0.03%)	90 (0.03%)	19 (0.0%)	χ2(df = 1) = 1.46; *p* = 0.02260
Medication induced disorder	445 (0.1%)	326 (0.1%)	119 (0.2%)	χ2(df = 1) = 5.19; *p* = 0.227
Other disorder	3,155 (1.0%)	2,542 (1.0%)	613 (0.9%)	χ2(df = 1) = 14.67; *p* = 0.0001
Intellectual or developmental disability	11,836 (3.8%)	9,559 (3.0%)	2,277 (0.7%)	χ2(df = 1) = 64.36; <0.0001
Multimorbidity	10,386 (3.3%)	7,550 (2.4%)	2,836 (0.9%)	χ2(df = 1) = 967.09; *p* < 0.0001
Health instability (CHESS 2+)	39,860 (12.8%)	26,261 (8.4%)	13,599 (4.4%)	χ2(df = 1) = 3712.76; *p* < 0.0001
Cognitive impairment				χ²(df=1)=1151.22; *p*<0.0001
Intact (CPS = 0)	214,525 (68.7%)	170,423 (70.2%)	44,102 (63.4%)	
Borderline intact-mild impairment (CPS = 1,2)	71,309 (22.8%)	52,994 (21.8%)	18,315 (26.4%)	
Moderate or worse impairment (CPS = 3+)	26,576 (8.5%)	19,471 (8.0%)	7105 (10.2%)	

### Prevalence of pain

Approximately 22.2% (N = 69,529) of people experienced pain. The characteristics of those with and without pain were statistically significantly different for almost all variables considered (Table 1). Most notably, those in pain were older (e.g., 21.2% aged 65+ vs 14.2%), more often female (54.1% vs 47.0%), more often partnered (33.6% vs 28.4%), more often self-reported trauma (16.7% vs 10.0%), more often had non-intact cognition (36.6% vs 29.8%), but fewer had psychotic disorders (10.7% vs 17.6%) or health instability (4.4% vs 8.4%).

### Recognition of pain management needs

Among the 69,529 individuals who reported experiencing pain, 49.0% (n = 34,069) had been offered, received, or were scheduled to begin receiving pain management.

Table 2 shows that, at the bivariate level, all but the following factors were significantly associated with recognition of pain management needs: age 65+ years (*p* = .4155); sex (*p* = .5250); being admitted from “other” setting (*p* = .3014); consumed 2-4 drinks in single sitting (*p* = .0634); use of hallucinogens (*p* = .5752); and obsessive compulsive (*p* = .6095), dissociative (*p* = .4716), elimination (*p* = .6292), sleep (*p* = .3500), sexual (*p* = .1549), gender (*p* = .3842), impulse (*p* = .1341), paraphilic (*p* = .0522), and other (*p* = .0908) disorders.

**Table 2.** Results of unadjusted and adjusted regressions.

Variables	OR (95% confidence interval)	
	Unadjusted	Adjusted
Age (ref = under 25)		
25–44 years	1.23 (1.16–1.30)	1.16 (1.10–1.24)
45–64 years	1.25 (1.18–1.33)	1.22 (1.15–1.30)
65+ years	0.98 (0.92–1.04)	1.00 (0.93–1.07)
Female (ref = male)	1.01 (0.98–1.04)	1.03 (0.99–1.06)
No partner (ref = partnered)	1.05 (1.02–1.09)	1.04 (1.00–1.07)
Admitted from (ref = private home)		
Congregated living	0.74 (0.68–0.81)	0.90 (0.82–0.98)
Hospital setting	0.73 (0.70–0.75)	0.81 (0.78–0.84)
Homeless	0.86 (0.78–0.94)	0.82 (0.74–0.90)
Correctional facility	0.62 (0.52–0.73)	0.72 (0.61–0.85)
Other	0.95 (0.86–1.05)	0.96 (0.87–1.06)
Made financial trade-offs in the last month (ref = no)	1.37 (1.29–1.5)	1.19 (1.11–1.26)
Previous psychiatric admissions (ref = none)		
1–2	0.94 (0.91–0.97)	0.97 (0.94–1.00)
3+	0.84 (0.80–0.89)	0.88 (0.83–0.93)
Highest # alcoholic drinks in single sitting (ref = 0)		
1	1.12 (1.04–1.20)	1.04 (0.97–1.13)
2-4	1.05 (0.10–1.11)	0.97 (0.92–1.02)
5+	1.29 (1.24–1.35)	1.10 (1.05–1.15)
Substance use in last week		
Inhalants	0.77 (0.62–0.95)	0.67 (0.53–0.83)
Hallucinogens	1.04 (0.91–1.19)	0.88 (0.76–1.02)
Cocaine	1.26 (1.20–1.33)	0.98 (0.92–1.05)
Stimulants	1.20 (1.12–1.28)	0.92 (0.85–0.99)
Opiates	1.92 (1.83–2.01)	1.75 (1.66–1.85)
Cannabis	1.10 (1.06–1.14)	0.98 (0.94–1.02)
Any substance	1.30 (1.26–1.35)	--
Reports trauma	1.679 (1.61–1.75)	1.48 (1.41–1.54)
Psychiatric diagnoses		
Neurodevelopmental disorders	0.83 (0.72–0.95)	0.88 (0.75–1.03)
Psychotic disorders	0.67 (0.64–0.71)	0.70 (0.64–0.76)
Bipolar disorders	0.78 (0.73–0.82)	0.77 (0.70–0.85)
Depressive disorder	0.95 (0.91–0.99)	0.84 (0.77–0.91)
Anxiety disorder	1.12 (1.05–1.19)	0.96 (0.87–1.06)
Obsessive compulsive disorder	0.95 (0.79–1.15)	0.89 (0.72–1.10)
Post-traumatic stress disorder	1.37 (1.28–1.46)	1.11 (1.01–1.23)
Dissociative disorder	0.83 (0.49–1.39)	0.74 (0.43–1.26)
Somatic disorders	1.32 (1.06–1.66)	1.23 (0.97–1.56)
Eating disorders	1.30 (1.08–1.56)	1.00 (0.81–1.23)
Elimination disorders	1.24 (0.52–3.00)	1.29 (0.53–3.14)
Sleep disorders	1.19 (0.82–1.73)	1.10 (0.75–1.62)
Sexual disorders	0.38 (0.10–1.44)	0.36 (0.09–1.37)
Gender disorders	0.72 (0.34–1.51)	0.81 (0.38–1.74)
Impulse disorders	0.83 (0.65–1.06)	0.85 (0.65–1.11)
Substance use disorders	1.37 (1.32–1.43)	1.25 (1.15–1.35)
Neurocognitive disorders	0.88 (0.82–0.95)	1.02 (0.91–1.13)
Personality disorders	0.85 (0.79–.91)	0.77 (0.69–0.85)
Paraphilic disorders	0.36 (0.13–1.01)	0.30 (0.11–0.86)
Other disorders	0.87 (0.73–1.02)	0.85 (0.71–1.01)
Medication induced disorders	0.60 (0.41–.87)	0.60 (0.41–0.89)
Intellectual/Developmental disability (IDD)	0.77 (0.71–0.84)	0.85 (0.78–0.93)
Multimorbidity	1.02 (0.93–1.12)	0.99 (0.90–1.10)
Health instability (ref = none)	1.34 (1.29–1.39)	0.73 (0.70–.76)
Cognitive impairment (ref = intact)		
Borderline intact-mild impairment	1.05 (1.13–1.09)	1.12 (1.08–1.16)
Moderate or worse impairment	0.93 (0.88–0.97)	1.07 (1.01–1.14)

Examination of multicollinearity revealed that use of any substance in the last week had a VIF of 6.03 with use of cannabis in the last week, exceeding the threshold of 5. As a result, any substance was excluded from the multivariate model to improve stability and interpretability.

In the multivariate model, several factors were associated with higher odds of recognition of pain management needs, including: ages 25-44 years (OR = 1.16, 95% CI = 1.10-1.24) and 45-64 years (OR = 1.22, 95% CI = 1.15-1.30); having no partner (OR = 1.04, 95% CI = 1.04-1.07); having made a financial trade off in the previous month (OR = 1.19, 95% CI = 1.11-1.26); having 5+ drinks in a single sitting (OR = 1.10, 95% CI = 1.05-1.15); use of opiates (OR = 1.75, 95% CI = 1.66-1.85); reporting trauma (OR = 1.48, 95% CI = 1.41-1.54); post-traumatic stress (OR = 1.11, 95% CI = 1.01-1.23) and substance use (OR = 1.25, 95% CI = 1.15- 1.35) disorder diagnoses; and borderline intact to mild (OR = 1.12, 95% CI = 1.08-1.16) or moderate or worse cognitive impairment (OR = 1.07, 95% CI = 1.01-1.13). Conversely, the following were associated with lower odds of recognition of pain management needs: admission from a congregated (OR = 0.90, 95% CI = 0.82-0.98), hospital (OR = 0.81, 95% CI = 0.78-0.84), or correctional (OR = 0.72, 95% CI = 0.61-0.85) setting; homelessness (OR = 0.82, 95% CI = 0.74-0.90); 3+ psychiatric admissions in the last 2 years (OR = 0.88, 95% CI = 0.83-0.93); use of inhalants (OR = 0.67, 95% CI = 0.53-0.83) or stimulants (OR = 0.92, 95% CI = 0.85-0.99) in the last week; psychotic (OR = 0.70, 95% CI = 0.64-0.76), bipolar (OR = 0.77, 95% CI = 0.70-0.85), depressive (OR = 0.84, OR = 0.77-0.91), personality (OR = 0.77, 95% CI = 0.69-0.85), medication-induced (OR = 0.60, 95% CI = 0.41-0.89), and paraphilic (OR = 0.30, 95% CI = 0.11-0.86) disorders; intellectual or developmental disability (OR = 0.85, 95% CI = 0.78-0.93); and health instability (OR = 0.73, 95% CI = 0.70-0.76).

The multivariate model had a −2 Log likelihood of 93598.75, compared to 96334.06 for the intercept-only model. This improvement in model fit was statistically significant (*p* < 0.001), indicating that the covariates improved the model. However, the model demonstrated limited discriminative ability, with a Concordance statistic (C-statistic) of 0.611, indicating that it was able to distinguish between those who had and did not have recognized pain management needs slightly better than chance. Although the C-statistic is greater than 0.5, it remains below the commonly accepted threshold of 0.7 for acceptable discrimination. Additionally, AIC and SC values for the full model (AIC = 93704.75; SC = 94189.64) were lower than for the intercept-only model supporting the model fit. Additional explanatory variables and model refinement may be required to improve discrimination.

## Discussion

The present study offers a system-wide view of recognition of pain management needs in psychiatric care, reporting on over 300,000 unique individuals who have received inpatient psychiatric services in Ontario, Canada. Pain was present among just over 20% of the population, and fewer than half of those with pain were offered, scheduled, or received pain management.

There were more individuals with pain who reported trauma and had a diagnosis of PTSD compared to those without pain. Both reported trauma and a diagnosis of PTSD were significantly associated with increased likelihood of having recognized pain management needs in the multivariate model. This association was not surprising, as chronic pain and PTSD commonly co-occur; pain can trigger or worsen symptoms of PTSD and PTSD can worsen the experience and management of pain (Feldman et al., 2014; Gasperi et al., 2021). Additionally, individuals with pain who have a history of trauma or PTSD often experience poorer functional outcomes, lower quality of life, heightened psychological distress, and reduced responsiveness to medical treatments (Gaetz 2017; Gasperi et al., 2021). These challenges may be associated with more frequent use of healthcare services as they seek relief and support.

The present study also found higher odds of recognition of pain management needs among those with substance use disorders, those who had consumed five or more drinks in a single sitting in the 2 weeks prior to the assessment, and those who had consumed opioids in the previous week. Maleki and colleagues (2019) reported that alcohol use and chronic pain often co-occur, and De Aquino and colleagues (2024) proposed that episodic binge drinking may be used to alleviate chronic pain. Opioid use was also strongly associated with higher odds of recognition of pain management needs. Current literature finds a mixed review about the association of opioid use and receipt of pain management, with large variations between illicit and prescribed use. Chang and Compton (2013) found that opioid prescription for those with mental illness provides a unique challenge to healthcare providers to avoid adverse effects. However, the current study found higher odds of having recognized pain management needs. Given that the assessment does not differentiate between prescribed and illicit substance use, it is possible the opioid use identified could reflect the form of pain management received, rather than illicit use. The study also found that use of inhalants and stimulants in the week prior to the assessment were associated with decreased odds of recognition of pain management needs. This association may be explained by previous research suggests that pain in individuals with active substance use may be deprioritized or dismissed, despite guidelines emphasizing the importance of addressing pain and harm reduction (Sowicz et al., 2022; Woodward and Braunscheidel 2023; Wyse et al., 2021).

This study further revealed a range of psychiatric diagnoses, including psychotic, bipolar, depressive, personality, paraphilic, and medication-induced disorders were associated with significantly lower odds of recognition of pain management needs. These findings are consistent with existing literature, which underscore that mental illness may act as a barrier to equitable pain management, which can lead to poorer health outcomes and reduced quality of life (Martinello and Matthews 2015; Udo and Gash 2012). In general, individuals with mental illness, are at increased risk to experience physical health problems and a high burden of pain yet often are less likely to receive pain management than their counter parts (Carrasco-Picazo et al., 2023; De La Rosa et al., 2024). Particularly, individuals with bipolar disorder have been found to have a lower likelihood of receiving pain management despite their elevated risk for poor health outcomes and reduced life expectancy (Garrido et al., 2017; Viprey et al., 2021). Stigma, clinician hesitancy, and systemic inadequacies in care coordination have been identified as possibly contributing to such findings (Stubbs et al., 2014).

This study also found that many different covariates considered were significantly associated with recognition of pain management needs. Our study’s findings related to age and sex differed from previous literature. More specifically, we found that pain was most prevalent among those 45–64 years, while the literature often cites older adults 65+ years as the most affected group (Kaye et al., 2010). We also found that there was no significant difference in recognition of pain management needs between adults aged 65+ and the reference group (i.e., under 25 years), though those aged 25–64 had significantly greater odds of having recognized pain management needs. This may reflect differing levels of communication ability, advocacy, or provider bias across age groups (Stubbs et al., 2014). Females comprised the majority (54.1%) of individuals reporting pain, however, pain in females may be underreported or under documented in clinical settings. For instance, Guzikevits et al. (2024) found that female patients’ pain scores were 10% less likely to be recorded in emergency department data despite reporting similar pain levels as men. Sex, however, was not significantly associated with pain management in the multivariate model.

Cognitive impairment was found to be associated with higher odds of recognition of pain management needs. This could potentially imply a growing focus on individuals with cognitive impairment, given their known status as an underserved group. However, the presence of an intellectual or developmental disability was significantly associated with lower odds, even after controlling for presence of cognitive impairment. This aligns with known disparities in pain management in this population (El-Tallawy et al., 2020).

Finally, the prevalence of health instability, as measured by CHESS, was lower among those who reported pain, and while it was associated with higher odds of recognition of pain management needs at the bivariate level, it was associated with lower odds in the multivariate model. This may indicate that individuals with higher CHESS scores may have multiple competing health issues that shift clinical focus away from presence of pain and need for pain management (Hirdes et al., 2003). There have long been recommendations for better integration of primary care and psychiatric care (Sowers et al., 2016), including embedded primary care in mental health settings (Maragakis et al., 2016). The current results highlight the need to prioritize creation of integrated care pathways in inpatient psychiatric settings to ensure that those experiencing health instability receive both holistic and appropriate care.

The present study utilizes population level data with over 300,000 unique individuals in Ontario; a key strength lies in the use of a large dataset which provides compressive information across multiple domains. However, due to the size of this dataset, it may also detect statistically significant associations that may have little clinical or practical significance, potentially leading to overinterpreting the findings. Substance use was assessed only for the previous 7 days and did not differentiate between prescribed and illicit use, an important limitation, particularly for opioid use. Multimorbidity was defined using a narrow set of available diagnoses, very likely resulting in an underestimation of its prevalence. Furthermore, dichotomizing and categorizing variables (e.g., age, CHESS, and cognitive performance scales) improved interpretability but reduced data granularity and masked distinctions in severity. Despite adjusting for many factors, model fit results suggest that unmeasured explanatory variables remain. While it was not the goal of this study to develop a full model predicting recognition of pain management needs, future studies are needed in this area. Finally, given that the long period covered by the data (i.e., 2005 to 2023), it would be important for future studies to examine whether the associations identified over the full period are replicated looking at specific periods of time – for example, during the COVID-19 pandemic.

## Conclusion

The present study provides insights into the disparities into recognition of pain management needs among individuals receiving inpatient psychiatric services in Ontario. It not only adds to the literature on the disparities experienced by persons with mental illness in terms of pain and pain management, but it also shows that some of these patterns continue to exist in specialized psychiatric care settings. Despite the high prevalence of pain, fewer than half of those reporting pain had been offered or received pain management, revealing a significant gap in care. Findings indicate that personal, social, functional, clinical, and service use factors are linked to lower odds of recognition of pain management needs, while reported trauma, recent opioid use, and certain socioeconomic indicators are associated with higher odds. These findings suggest that pain management may be influenced by intersecting vulnerabilities and visibility within mental health settings. An integrated, person-centered approach is needed to address the unique needs of this underserved population. Future research should explore these associations longitudinally and incorporate qualitative or mixed methods to further understand patient experiences, decision-making, and unmet needs.

## Data Availability

The data are made available to interRAI Fellows (Martin) for research use under an existing license agreement interRAI has with the Canadian Institute for Health Information; note that the agreement is for research only, not commercial use. Students working under the supervision of an interRAI Fellow can apply for free access to the data but are subject to terms of use. As part of interRAI’s agreement with the Canadian Institute for Health Information, the data may not be transmitted to third parties; therefore, the data used in this study cannot be made available to others. Those interested in using the data can apply directly to the Canadian Institute for Health Information for access.
